# A Novel Approach to the Holistic 3D Characterization of Weld Seams—Paving the Way for Deep Learning-Based Process Monitoring

**DOI:** 10.3390/ma14226928

**Published:** 2021-11-16

**Authors:** Maximilian Schmoeller, Christian Stadter, Michael Karl Kick, Christian Geiger, Michael Friedrich Zaeh

**Affiliations:** Institute for Machine Tools and Industrial Management, TUM Department of Mechanical Engineering, School of Engineering & Design, Technical University of Munich, Boltzmannstr. 15, 85748 Garching, Germany; christian.stadter@iwb.tum.de (C.S.); michael.kick@iwb.tum.de (M.K.K.); christian.geiger@iwb.tum.de (C.G.); michael.zaeh@iwb.tum.de (M.F.Z.)

**Keywords:** non-destructive testing, weld seam contour, microfocus computed tomography, laser beam welding, Deep Learning

## Abstract

In an industrial environment, the quality assurance of weld seams requires extensive efforts. The most commonly used methods for that are expensive and time-consuming destructive tests, since quality assurance procedures are difficult to integrate into production processes. Beyond that, available test methods allow only the assessment of a very limited set of characteristics. They are either suitable for determining selected geometric features or for locating and evaluating internal seam defects. The presented work describes an evaluation methodology based on microfocus X-ray computed tomography scans (µCT scans) which enable the 3D characterization of weld seams, including internal defects such as cracks and pores. A 3D representation of the weld contour, i.e., the complete geometry of the joint area in the component with all quality-relevant geometric criteria, is an unprecedented novelty. Both the dimensions of the weld seam and internal defects can be revealed, quantified with a resolution down to a few micrometers and precisely assigned to the welded component. On the basis of the methodology developed within the framework of this study, the results of the scans performed on the alloy AA 2219 can be transferred to other aluminum alloys. In this way, the data evaluation framework can be used to obtain extensive reference data for the calibration and validation of inline process monitoring systems employing Deep Learning-based data processing in the scope of subsequent work.

## 1. Introduction

For the qualification of welding processes in industrial production, both the quality of the weld seams and the process stability are of essential interest. For evaluating these properties, metallographic methods such as longitudinal or lateral cross-sections of the weld seam samples or X-ray imaging methods are commonly used. The test criteria cover a wide range of aspects, including internal defects such as cracks and pores, as well as the weld interface and the weld seam cross-section [1]. To ensure the accuracy of the evaluation, the measurement results must typically include a statistical verification to reflect the temporal fluctuations of the test criteria throughout the production process. 

At present, no test procedure allows the validation and correlation of the test criteria mentioned above based on one evaluation method. As a result, a combination of different examination methods that require a high empirical expertise has to be applied for a comprehensive evaluation of the weld seams regarding geometric characteristics and internal defects. In particular, for the qualification and parameterization of inline process monitoring systems, which allow an evaluation of the weld seam quality during the production process without a subsequent inspection, temporally and spatially correlated reference data are required [2]. Therefore, it is of central importance that a scale-bridging set of information on weld seam characteristics can be obtained using suitable methods to enable an evaluation of the correlations between the test criteria, e.g., the mutation of the weld pool cross-section induced by the formation of process pores. 

In the context of this work, an approach based on industrial microfocus X-ray computed tomography (µCT) for the holistic evaluation of weld seam characteristics concerning all geometrically quantifiable properties of weld seams is presented. In addition, a method for the temporal and spatial correlation of the measured properties with the signals of inline process monitoring systems during deep penetration laser beam welding is proposed. This will provide the basis for a significant increase in the accuracy of inline weld seam evaluation and testing. Thus, the way is paved for the use of Deep Learning data processing approaches with a methodology for the acquisition of high-quality and extensive reference data [3].

## 2. State of the Art

### 2.1. Offline Characterization of the Weld Seam Geometry and Weld Defects

Several methods have been established for the offline characterization of weld seams, which differ from each other in the type of quality features, i.e., weld seam defects, that can be detected. In the following, methods for the characterization of metallic weld speci-mens are presented in general, whereby, with regard to their use in industrial production, welding techniques of arc welding, high-energy beam welding and solid-state welding are considered in particular. 

Metallographic analyses as a class of destructive testing methods offer the possibility of analyzing the microstructure, the weld seam geometry and internal defects in sectional planes through the weld seam [4]. Internal seam defects such as pores, cracks and the microstructure can be evaluated using optical microscopy images [5]. However, the effort required to prepare the joint specimens and the challenges of ensuring the desired position of the sectional planes, especially in the case of micrograph specimens parallel to the direction of the weld seam, often allow only an evaluation at selected planes and limit the informative value.

For non-destructive analyses, *ultrasonic testing* is a common method for industrial inspection purposes. It can be used to detect internal weld seam defects. While many process variants exist, most aim at volumetric integrity testing, which allows the detection of irregularities such as pores [6]. Application research on ultrasonic phased array technology for weld seam inspection could prove the ability to detect inclusions and pores which were deliberately triggered during multi-layer multi-pass welding [7]. Ultrasonic testing was also applied to assess porosity and tiny voids in friction stir welded joints [4].

X-ray computed tomography (CT) is well established in various industries for the examination of internal properties of structural components that affect the attenuation of X-rays and is thus a method that can be applied to inspect weld seams [8]. The radiography of a sample from different angles and the analysis of the attenuation of X-ray radiation allow three-dimensional (3D) scans. For the analysis of weld seams, the superficial weld seam dimensions can be measured, and the weld seam quality can be assessed by determining the porosity [9]. By utilizing gamma-ray CT, it could be shown that weld discontinuities in steel pipes down to 0.3 mm [10] and reductions in the effective thickness of welds due to defects on the order of 20% can be detected [11]. 

For welding in an overlap configuration with a pronounced air gap between the upper and lower joining partners, the cross-sectional diameter of the joint was determined based on CT scans [12]. X-ray CT was also deployed for the 3D inspection of butt welds in coil tube manufacturing. The feasibility of evaluating the shape of the weld seam root and the cap surfaces to identify weld flaws was demonstrated [13]. Analyses of void structures and a segmentation of CT scans could reveal 3D models of gas-filled defects in friction stir welds [14].

Microfocus X-ray computed tomography (µCT) allows resolutions with voxel dimensions down to a few micrometers by reconstructing several thousand radiographic images. Thus, defects in railheads could be visualized and, on this basis, a 3D model of crack structures was created [15]. Microstructural and CT analyses could reveal voids in extruded aluminum weld seams [16]. X-ray dark-field CT was used to investigate microstructural changes induced in macroscopically homogeneous materials during friction stir welding. The imaging technique provided three-dimensional information about the microstructure of the welds on a macroscopic scale [17].

All test methods used for weld seam characterization can provide valuable information about individual quality characteristics but are suitable mostly only to a very limited extent for a comprehensive inspection. With existing approaches to weld seam inspection, only individual quality features can be evaluated. Therefore, the choice of the method is strongly determined by the respective target value and is usually only applicable to other weld seam characteristics within a highly constrained range.

### 2.2. Online Process Monitoring in Laser Beam Welding

For process monitoring during welding, a wide range of target values and methods have been investigated. To a large extent, optical methods, which will be considered and discussed with view to different objectives in detail below, are used [2]. The *weld surface inspection* can provide information on the process stability and the superficially detectable weld seam formation. A composite sensor system based on active and passive optical sensors was developed to monitor the weld pool in gas metal arc welding. (Passive sensors can be used only to detect radiant energy if the process being observed is emitting radiant energy. Active sensors provide a dedicated energy source for illuminating the observed process.) The obtained information was used to reconstruct the melt pool surface [18]. A binocular vision sensing system could be used to reconstruct the melt pool geometry based on a passive vision measurement by a single camera [19]. In general, the high intensities in the area of the process zone during laser beam welding make the use of optical systems challenging. Low-coherence interferometry, as the first method to allow dimensional measurements directly in the process zone, allows high-precision in-situ measurements of the weld seam topology [20].

Low-coherence interferometry also allows for a direct weld depth measurement during laser beam welding. It could be demonstrated that the interferometric measuring principle, applied coaxially to the laser beam, enables a measurement of the capillary depth during deep penetration welding [21]. In addition to the determination of the penetration depth, the quality of the weld seam surface concerning the occurrence of irregularities in the topography could be determined based on the measured capillary depth [22].

Internal defect detection is possible only to a limited extent during welding to date. Online X-ray observations can provide insights into the formation of process pores during laser beam welding [23], though their application in industrial production is limited due to the complex system technology required. Although many sensor technologies have been tested, the reliable detection of cracks and detection of pores is only possible to a very limited extent.

Process monitoring systems are designed very specifically and are usually built individually for a particular application and a specific target variable. The acquisition of temporally and spatially correlated, reliable data for process adjustment and quality assurance is a significant challenge.

## 3. Objectives and Approach

**Figure 1:**
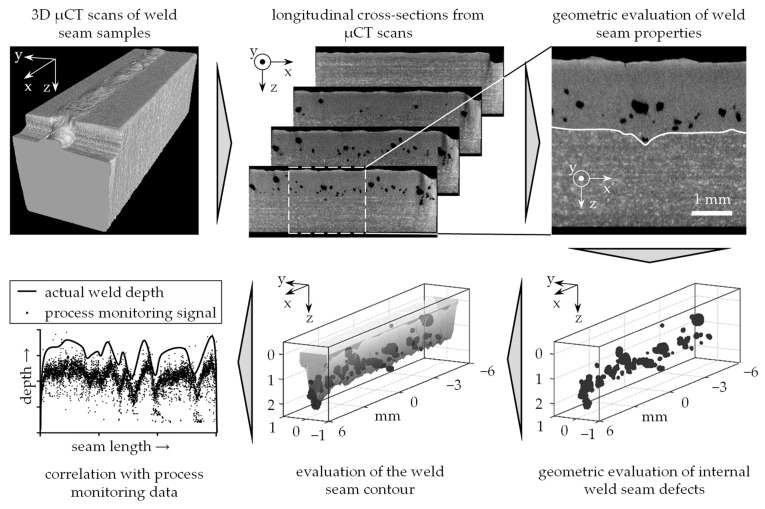
Approach for the development of a 3D characterization method of weld seams concerning internal defects and the weld seam contour as well as for the correlation of the measurement results with inline process monitoring data.

This paper presents an approach that allows to capture and geometrically quantify all properties required to evaluate the quality of a weld seam. For data acquisition, industrial microfocus X-ray computed tomography is used, whereby the measurement parameters are optimized so that internal defects such as pores and cracks can be resolved. In addition, it is possible to detect changes in the metallurgical microstructure inside the component caused by the melting of the material during deep penetration laser beam welding. Figure 1 shows an exemplary data set including all geometrically quantifiable weld seam properties such as a 3D representation of the weld seam contour, of cracks and of pores.

With the help of the measuring method based on microfocus X-ray computed tomography, it is possible to obtain a three-dimensional image of the entire weld seam that contains information on the weld seam geometry and the position of defects in the weld seam contour and in the component. The distribution and the sizes of pores and cracks within welds, which in this paper were induced during deep penetration laser beam welding, can be obtained from this information. Additionally, information about the weld depth and the weld seam cross-section can be obtained. The relationships between process instabilities and the weld seam geometry can be investigated by applying subsequent data evaluation steps. For the first time, a complete characterization of weld seams is possible using a 3D data set, which enables novel insights into the relationships between process parameters, process stability and the resulting weld seam properties.

The geometric measurement data from the weld seams subsequently can be compared with the data from inline process monitoring systems, which are recorded during the production of the evaluated weld samples. As a first step towards this, a procedure to compile temporally and spatially correlated data sets is presented in this paper. The process monitoring data can then be examined for information on weld defects and process instabilities. These temporally highly resolved data sets form the basis for advanced interpretation methods for sensor signals employing Deep Learning approaches.

## 4. Materials and Experimental Set-Up

Aluminum alloys pose a comprehensive challenge with respect to weldability. Due to their physical properties, aluminum materials are generally susceptible to weld seam defects and process instabilities. Therefore, reliable quality assurance methods capable of being integrated into an automated processing system are needed to ensure the required characteristics of high-quality welds for the broad and efficient use of aluminum in lightweight constructions. Thus, the focus of this paper is on laser beam welding of aluminum alloys. For the development of the characterization method, a material was selected that allows the detection of microstructural changes caused by the welding process by means of radiographic testing. Five different aluminum alloys, which differed in their composition, heat treatment and manufacturing process, were investigated within the preliminary experiments (cf. Figure 4). The materials were evaluated to determine the extent to which changes in the microstructure caused by welding resulted in a detectable change in the attenuation of the radiation from the µCT, according to the investigations of Schaff et al. [17]. The alloy AA 2219 was selected for further analyses as it allowed a complete visuali-zation of the weld geometry inside the specimens. The material range was limited to alloys with high strength and high technical relevance for industrial production. The material data are summarized in Table 1.

**Figure 2:**
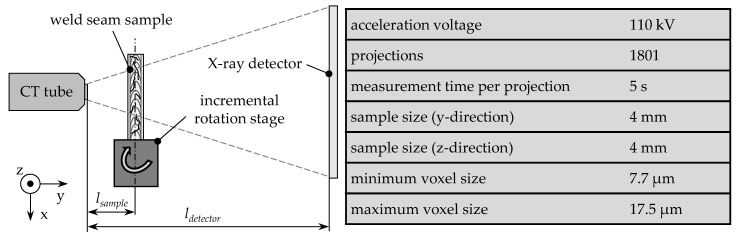
Schematic representation of the measurement set-up and measurement parameters for the acquisition of µCT images of weld seam samples.

A microfocus X-ray computed tomography (µCT) system of the type Xradia Versa 500 from ZEISS was used for the radiographic inspection of the weld seam samples (cf. Figure 2 for the measurement parameters). The system acquired three-dimensional images of a specimen with a voxel resolution in the single-digit micrometer range. The speci-men was mounted on a rotatable fixture. During the measurement, the sample was rotated a total of 360 degrees in fine angular increments, whereby a radiographic picture of the sample was taken in each angular position. Individual images are referred to as projections. A three-dimensional data set was generated by the so-called reconstruction. The resolution of the individual voxels is determined by the distance of the sample to the radiation source $l_{sample}$ and by the distance of the detector screen to the radiation source $l_{detector}$. For the analysis of samples, the measuring radiation is generated in the CT tube and guided to the sample by means of the acceleration voltage. When passing through the sample, the radiation is attenuated, while the optical resistance of a sample determines the degree of attenuation. Thus, X-rays are attenuated less when passing through a porous section in the weld metal compared to when passing through a solid metal body. The beam intensity is subsequently measured on the detector screen and forms the basis for reconstructing the 3D data. Figure 2 shows the basic structure of a µCT system and the parameters of the system used.

**Figure 3:**
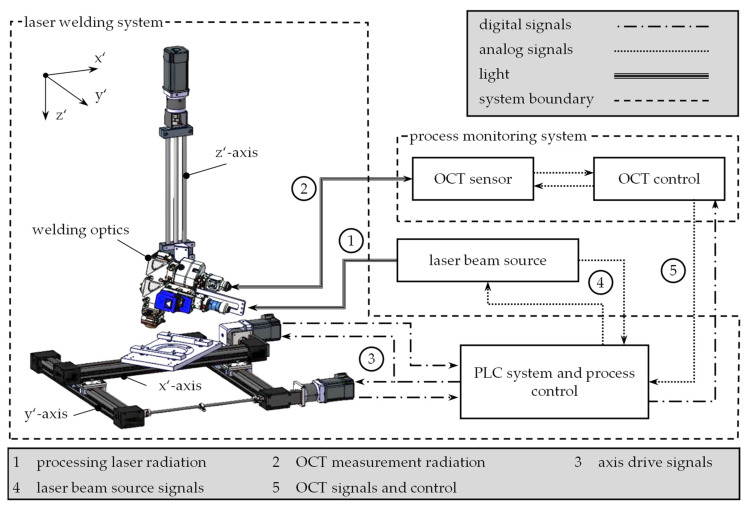
Structure of the laser beam welding system with the data and light streams between the system components.

To prepare the weld seam samples, a laser beam welding system was used, which was specially designed for the time synchronization between signals of a process monitoring system and the control signals of all process-influencing devices. For this purpose, all weld specimens were provided with a precisely positioned notch, which could be clearly detected and localized in both the µCT images and the process monitoring data (cf. Figure 1). A fixture consisting of a base plate and a cover plate was used to position the welding specimens. The bottom plate contained a recess for the test specimens, which were cut to 115 mm length and 4 mm width from 4 mm material thickness by waterjet cutting. The cover plate was fixed by means of clamping levers, which allowed the weld specimens to be positioned uniformly across all tests and created comparability between the tests. At the core of the set-up, there was the programmable logic controller (PLC), which processed all measurement and control signals and provided them with a time stamp. Inside the PLC, the process control algorithm was implemented, which allowed a real-time adaptation of the process parameters to the measured control variables. Within the scope of the experiments, a fixed optics system of the type YW52 from Precitec GmbH & Co. KG, Gaggenau, Germany, was used. The optics was integrated into a 3-axis CNC system, allowing to adjust the distance of the focal point to the component as well as to perform the feed movement of the component relative to the fixed working spot of the optics. The fixed optics was positioned with an angle of incidence of 10° in a piercing configuration. A focal diameter of 100 µm at a focal length of 200 mm was utilized for the experiments [24]. Three synchronous servo motors were used for the feed motions, which can send their respective position to the control in real-time. Thus, the actual feed rate of the component in the x’y’-plane could be determined from the obtained data. A continuous-wave multi-mode fiber laser of the type FL080 from Coherent Inc. provided the near-infrared laser radiation, which was guided to the optics by a fiber optic cable with a core diameter of 100 µm. The laser beam source was integrated into a control loop, within which it received a power target value from the PLC and reported back the actual power. An inline process monitoring system based on Optical Coherence Tomography (OCT) was integrated into the welding system, allowing to measure the distance between the processing optics and a specific spot on the component surface at an acquisition rate of 70 kHz. For this purpose, a measuring laser beam was coupled coaxially to the processing beam within the optics. The measurement position was not changed over all experiments, as the focus of the investigations was on the qualification of µCT for the reconstruction of weld seams, and thus, influences of the welding parameters and the material on the OCT signal characteristics were not considered in this study. The OCT sensor sent the evaluated distance signal to the PLC, which triggered the process monitoring and synchronized all recorded process variables. Figure 3 shows the schematic structure of the laser beam welding system and the information flows that were transmitted in the form of light, digital signals or analog signals.

## 5. Methodology

**Figure 4:**
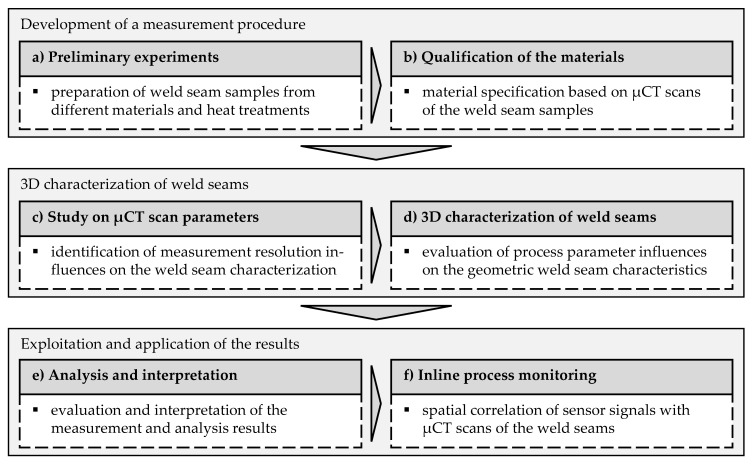
Methodological approach for the development of a 3D weld seam characterization method to obtain reliable reference data with temporal correlation to inline process monitoring data.

The methodology for developing a 3D weld seam characterization procedure can be divided into three steps. In the first step, an X-ray-based measurement procedure is quali-fied for the acquisition of radiographic raw data. Subsequently, the results are applied to develop an evaluation routine for the three-dimensional assessment of all geometrically quantifiable properties of weld seams. In a third step, the characteristics concerning their correlation with process parameters are evaluated and aligned with sensor signals from inline process monitoring systems. The overall goal is to obtain a detailed database of weld characteristics that can be used to significantly improve inline process monitoring systems. A significant challenge is to provide a reliable correlation between sensor signals and process results. A comprehensive and time-correlated reference to sensor signals enables the use of Deep Learning approaches. The overall methodology is shown in Figure 4.

In the following, the particular elements of the methodology are explained:

(a) Preliminary experiments: In a preliminary experimental study, laser beam welding tests are carried out on various aluminum alloys without varying the welding parameters. The aim is to produce specimens for preliminary tests with µCT inspection.
(b) Qualification of the materials: The µCT scans of the weld seam samples from the preliminary test series are evaluated and assessed with respect to their information content. For further investigations, the alloy AA 2219 is selected, allowing a comprehensive characterization of the geometric weld seam properties.
(c) Study on µCT scan parameters: In this step, an experimental study is carried out to evaluate the influence of the measurement parameters in µCT inspection on the quality of the resulting 3D images. The aim is to determine measurement parameters resulting in high contrast and detail quality of the images while maintaining the largest possible measurement volume.
(d) 3D characterization of weld seams: Initially, weld seam samples are prepared following a full factorial experimental design. The samples are scanned using µCT with the previously defined measurement parameters. Based on the results of the measurements, the influences of the welding process parameters on the formation of the weld seam contour and on the occurrence of internal seam defects are investigated.
(e) Analysis and interpretation: In this step, the individual result components are composed. For the first time, signal characteristics could be correlated with the real-time process parameters and weld seam defects, resulting in extensive information on the weld seam geometry. Extensive knowledge about the phenomena during the welding process can be gained from this.
(f) Inline process monitoring: The findings gained from the analysis of the weld seams are compared with measurement signals from inline process monitoring systems. For this, the signals are correlated spatially with the µCT images and examined for distinct features. In addition, a method is developed which allows the entire data set to be decomposed into individual temporal elements to create an extensive database as a platform for Deep Learning approaches for the evaluation of measurement signals.

## 6. Experimental Results

In this chapter, the results of the weld seam characterization and the correlation of process monitoring signals with geometric features of the weld seams are presented.

### 6.1. Influences of Process- and Measurement-Parameters on µCT Images

In the first step, the influence of the measurement parameters, which can be varied during the acquisition of the µCT images, on the quality of the reconstructed 3D data sets was examined. The resolution, expressed in the voxel size, could be adjusted by moving the sample on the axis between the X-ray tube and the detector in the µCT system (cf. Figure 2). A high resolution, i.e., a small voxel size, leads to a reduced size of the recorded sample section. The relation between the resolution and the observation area results in a conflict of targets. Therefore, the resolution should be determined in a way that all relevant geometric features can be assessed, and at the same time, the largest possible sample section can be recorded. Table 2 summarizes the voxel numbers contained in a data set and the corresponding dimensions of the measuring range for the variety of resolutions that were investigated within the preliminary studies.

**Figure 5:**
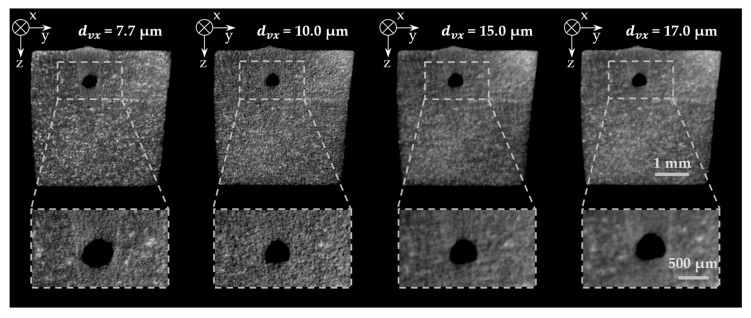
Evaluation of the influence of the measurement resolution on the differentiability of the microstructure of the base material and the weld seam for voxel sizes *d_vx_* between 7.7 µm and 17 µm using cross-sectional evaluations of the 3D µCT scans; material AA 2219, laser power 1.5 kW, feed rate 9 m/min.

Figure 5 shows cross-sectional images of a weld sample acquired with different voxel dimensions. Moreover, a relevant section of the image from the upper part of the cross-sectional images is enlarged, covering parts of the base material and the weld seam with an enclosed defect. In particular, the high-resolution images resulting from the reconstruction of the µCT scans show the fine-grained texture of the base material. In the weld seam, i.e., in the area melted during the welding process, a differing texture with a more homogeneous gray value distribution was observed. The finely resolved images revealed that the weld seam in its three-dimensional shape inside the sample could be identified very well, based on the change in the image texture. As a result, the voxel size for generating the images must be smaller than the size of the individual gray value regions to be distinguished. For the samples with a resolution of 7.7 µm and 10 µm (measured by the voxel size *d_vx_*), the condition was well satisfied, allowing a precise differentiation between the base material structure and the weld seam structure. At a resolution of 15 µm and coarser, relevant information about the weld seam geometry was lost as the voxel size is on the order of magnitude of the texture variations. Thus, adjacent areas with different gray values overlapped by a voxel, inducing reconstructed voxels to represent an average gray value of the detected microstructure areas.

As an additional criterion for selecting a suitable measurement resolution, the detectability of weld seam defects in the 3D scans was evaluated. The size of the pores induced by the welding process was typically on the order of several hundred microns. Cracks, in contrast, were observed to have dimensions on the order of a few microns. Due to the characteristically large-area impairment of the structural cohesion and the resulting weakening of the weld seam strength by cracks, it is essential that this defect class can be detected accurately. Therefore, despite the reduced spatial coverage of the sample, the highest possible resolution was selected. In the series of measurements presented, a voxel size of 7.7 µm proved to be well suited since all essential geometric characteristics were contained in the reconstructed 3D data. 

**Figure 6:**
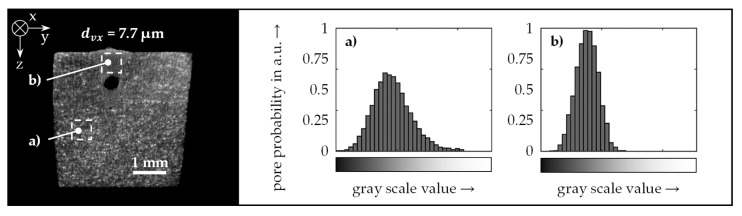
Gray value distribution of a cross-sectional evaluation of a µCT scan with a voxel size *d_vx_* of 7.7 µm for a representative area of the base material (**a**) and the weld seam (**b**); material AA 2219, laser power 1.5 kW, feed rate 9 m/min.

Figure 6 shows the results of further investigations of the image properties at the selected measurement resolution. Histograms were used to evaluate the gray value distributions in the area of the base material and the weld seam. It was evident that the position of the gray value maximum for the areas (a) and (b) was not affected by the welding process. However, the weld seam area contained significantly fewer bright voxels, which were characteristic for the texture of the base material. This observation confirmed that a reasonable distinction could be found between the base material and the melted area of the weld seam based on the texture of the CT scans, i.e., the microstructure of the sample.

### 6.2. Analysis of Process Pores

Pores mostly represent gas inclusions that remain in a weld seam after the solidification of the melt. In general, process and hydrogen pores can be distinguished. Process pores are caused by dynamic instabilities of the welding process, due to which gas, that cannot rise to the melt pool surface during solidification, is introduced and trapped in the melt. Characteristic for this type of defect are the heterogeneous shape and strongly fluctuating dimensions. Hydrogen pores are caused by hydrogen outgassing during the cooling of the melt during solidification due to a decrease in solubility in the liquid metal. They typically have much smaller dimensions than process pores and are usually spherical. In general, pores are disruptive points in the microstructure of welds and thus lead to a decrease in the strength of joints. Therefore, one of the objectives in the design of welding processes is to avoid porosity by adjusting the process boundary conditions [25].

**Figure 7:**
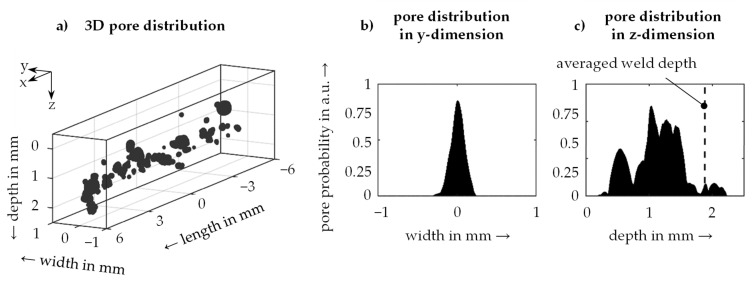
Characterization of porosity based on 3D measurements: (**a**) 3D distribution of process pores, (**b**) distribution of pores over the width and (**c**) over the depth of a representative weld seam; material AA 2219, laser power 1.5 kW, feed rate 9 m/min.

The 3D characterization method for weld seams presented within this paper allows for the first time to obtain comprehensive information about the relationships between pores and the weld seam geometry. Figure 7 shows on the left the pores recorded for an exemplary weld seam using a µCT scan. A classification dividing the entire data set into the classes pores and solid material was carried out for the evaluation. Referenced to the sample contour, the spatial distribution of the pores for the dimensions evaluated in cartesian dimensions was evaluated. For the analysis of the position, the probability of a voxel being assigned to the class pore in relation to a certain geometric dimension was used as a metric for position analysis. On the right side of Figure 7, the probabilities of the occurrence of pores over the weld width and the weld depth are presented. Based on the evaluations, it was found that pores occur predominantly in the center of the weld seam. Given the pore distribution in the depth direction, it was determined that the probability of a pore occurring on the weld surface and in the area of the average weld depth is low. The defects were mainly found in the lower half of the weld depth value range. This phenomenon can be attributed to the formation of process pores. While near-surface pores rise during the solidification of the melt and can thus outgas, deeper pores are trapped in the weld metal and remain in the form of voids.

### 6.3. Evaluation of the Weld Depth

**Figure 8:**
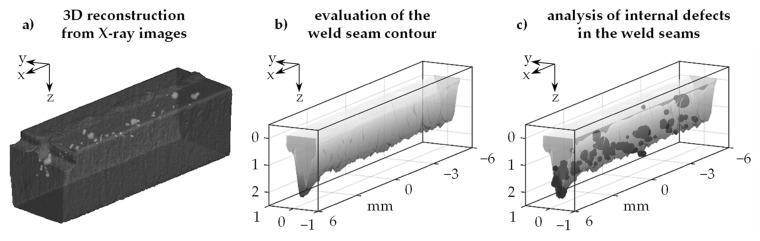
(**a**) 3D reconstruction of µCT scans of a weld seam; (**b**) evaluation of the weld seam contour; (**c**) examination of the correlations between the weld seam contour and internal defects; material AA 2219, laser power 1.5 kW, feed rate 9 m/min.

The weld depth is an essential strength-determining feature in the design of welding processes. Particularly in deep penetration laser beam welding, high weld depths of several millimeters can be achieved. Many technical applications require the high stability of the weld depth since fluctuations can lead to damage of the joining partners or an insufficient connection. Destructive metallographic test methods such as transversal and longitudinal cross-sections are usually used to determine the weld depth. The test results are often statistically verified to more deeply consider fluctuations due to process dynamics and disturbing influences. With the help of the presented characterization method, a complete 3D profile of a weld seam can be assessed for the first time. The generated data can be evaluated, for example, with respect to weld depth, which provides a continuous depth profile in the feed direction. Thus, errors, inaccuracies and missing information that cannot be avoided in characterizing the penetration depth by punctually applying metallographic methods can be excluded. Figure 8 shows a completely evaluated weld seam contour. Left hand side in the figure (a) is the 3D data set obtained from the reconstruction of the µCT data. In the middle (b), the internal weld seam boundary, which has a highly heterogeneous shape, is shown. On the right (c), the pores discussed in Section 6.2 are superimposed onto the weld contour. In the course of an image processing, the visibility of the image area of the solidified melt was improved. Through an image preparation method referred to as gray value transformation, a manipulation of the gray value areas of the pixels was performed, i.e., the application of point operations to the pixels of a cross-sectional image in the form of a transformation characteristic. Using the image processing method, the gray values of the pixels of the images were merely scaled. The improved visual properties could be attributed to the linear transformation of the pixels in the middle gray value range (brightness values of 107–146 with upper and lower limits of 0 and 255 of the images, respectively). The advantage of the complete 3D characterization of weld seams compared to metallographic sections is the significantly increased trustworthiness of the data and the subsequently derived information. When looking at micrographs, no statement can be made about the accuracy, only an estimation. The 3D representation of the weld seam geometry shows that the weld depth does not always reach its maximum value in the middle of the seam, which could be suggested by a punctually applied cross-section. Since cross-sections only allow a point-by-point evaluation of the weld seam dimensions and thus a very large number of metallographic micrographs have to be taken for a statistically reliable comparison with the 3D radiographs, an evaluation of the accuracy of the measurement method will be carried out within the scope of subsequent studies. In addition, 3D data sets can be used to analyze correlations between geometric changes in the weld seam cross-section and defects such as pores. Knowledge can be gained about the dynamic processes during welding and the effects on the weld seam geometry from this.

### 6.4. Correlation between µCT Images and Process Monitoring Sensor Signals

With the help of the presented approach, it was possible to characterize welds in AA 2219 samples three-dimensionally and to gain information about the weld seam geometry as well as internal defects in the sample. In addition, a laser welding system was developed that allowed to temporally correlate measurement signals from inline process monitoring sensors with the welding process. The following section discusses how the generated measurement data sets can be spatially correlated with the results of the weld seam characterization. This makes it possible for the first time to obtain completely coherent data sets that can be used to significantly increase the accuracy and information density in the interpretation of process monitoring signals. 

**Figure 9:**
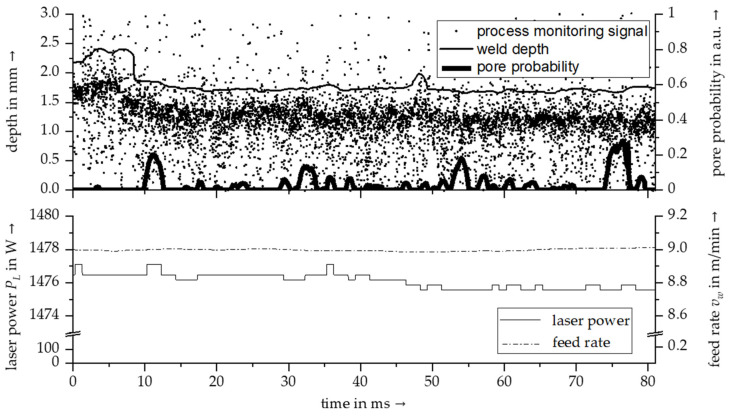
Temporal correlation of process monitoring signals (e.g., a keyhole depth measurement signal based on Optical Coherence Tomography), machine feedback data and the results of the 3D weld seam characterization concerning the weld depth and the distribution of pores; material AA 2219, laser power 1.5 kW, feed rate 9 m/min.

Figure 9 shows a correlated data set. The upper diagram illustrates the weld depth and the distribution of pores along the weld seam obtained from µCT scans. In addition, the raw signal of a keyhole depth measurement based on Optical Coherence Tomography is shown, which was recorded inline during the generation of the weld seam sample. The result of the OCT measurement is visualized as a point cloud that represents a high-resolution and time-discrete distance measurement. It can be clearly seen that the area of the OCT signal with the highest density in depth direction was below the real weld depth. This behavior is due to a melt layer surrounding the vapor capillary in deep penetration laser beam welding [26]. In addition to evaluating the capillary depth, the OCT signal can be utilized to assess correlations between the course of the weld depth and the process stability. The hypothesis that the course of the measured capillary depth correlates with the process stability during laser beam welding could already be confirmed in [22]. Therefore, in further investigations, it can be evaluated analogously to what extent a correlation between mutations in the OCT signal and the distribution of process pores can be found. In the lower part of Figure 9, the feedbacks of the laser welding system on the actual laser power and feed rate are shown. It can be seen that both sequences showed only slight fluctuations. It can be assumed that the observed process instabilities, which occurred during the welding process, were not a result of fluctuations in the process parameters; however, a more detailed investigation and evaluation has to be the subject of future studies. 

Machine Learning methods are often used to evaluate signals such as those recorded by process monitoring systems. However, to achieve high prediction accuracies, the methods have to be trained using accurate reference data, which are often difficult to obtain. In particular, the temporal and spatial correlation of samples and data sets is challenging given the inaccuracies of commonly used metallographic methods. In the presented approach, this challenge was mastered by using the measurement signals as a reference. The machine and sensor data were each provided with a time stamp. In order to correlate the µCT images, the welding samples were given a defined notch in the test preparation. During the welding process, this notch led to a characteristic change in the OCT signal of the keyhole depth measurement. On the basis of the measurement signal, the time at which the welding process passes through and exits the notch could thus be determined. This also allowed the time stamp to be assigned to the µCT scans and the data obtained from them. The recorded feed rate could also be used in combination with the data time stamps to calculate the position where the data was measured along the weld seam. 

This method allows high-quality and precise data sets to be obtained for training Machine Learning methods. The limitation in the temporal resolution is determined only by the frequency of the data recording, which was 1000 Hz for the presented laser welding system. This paves the way for data-driven approaches such as Deep Learning, which can achieve new performance levels using large amounts of training data compared to data processing approaches available for the evaluation of inline process monitoring data during welding so far.

## 7. Conclusions and Outlook

An evaluation method based on microfocus X-ray computed tomography (µCT) for the inspection of metallic specimens was developed to acquire a three-dimensional representation of welds, including all geometrically quantifiable specimen-internal features. As a result, extensive information on the weld geometry and internal defects could be obtained from the scans. By building on this, it is possible to reconstruct relationships between process parameters, dynamic fluctuations during the welding process and the weld seam quality utilizing a data set with unprecedented comprehensiveness and accuracy in future studies. 

The approach for the development of a 3D weld characterization method was divided into three main steps. In the first step, an X-ray-based measurement method was qualified for the acquisition of radiographic raw data. Subsequently, the results were used to design an evaluation routine for the three-dimensional assessment of the geometric properties of welds. Finally, the acquired geometric features were evaluated with respect to their relationship to process parameters and correlated with sensor signals from inline process monitoring systems. 

Welding tests on aluminum alloys with different microstructures, material compositions and manufacturing processes were carried out to qualify the measurement system. Based on the investigations of Schaff et al. [17], it was presumed that a significant change in microstructure induced by the welding process could lead to a detectable change of the attenuation of X-rays when passing through a sample. As part of a preliminary experimental series, weld specimens were examined using a µCT device with respect to material qualification. The measurements on the aluminum alloy AA 2219 allowed a complete visualization of the weld geometry inside the specimens. In addition, acquisition parameters of the µCT system were evaluated in terms of measurement resolution. With a voxel size, the three-dimensional equivalent of the pixel size, here of 7.7 µm edge length, a high contrast of the weld metal compared to the base material as well as a good detectability of seam defects such as cracks could be achieved. 

A methodology based on the 3D weld seam characterization results was established for the local and temporal correlation of the geometric weld seam features with the measurement signals from inline process monitoring systems such as an OCT sensor. In order to correlate the signals, a reference notch was added to the weld specimens before conducting the experiments. In the keyhole depth measurement signal of the OCT sensor, the notch yielded a characteristic pattern. Furthermore, due to the complete and temporally correlated closed-loop acquisition of all sensor signals and all axis positions and velocities, the notch, which was detectable in the OCT signal and the µCT images, could be used for referencing. Thus, comprehensive reference data for the weld depth and the characteristic geometric properties of the weld were obtained for the evaluation of sensor signals.

**Figure 10:**
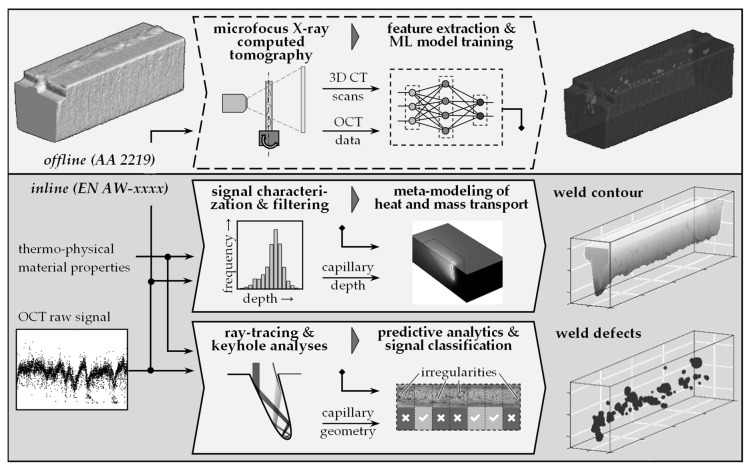
Approach for the transfer of the findings from the 3D characterization of welds based on AA 2219 to other materials using simulation and Machine Learning (ML) methods.

The presented measurement method can be used in subsequent investigations to obtain high-resolution and comprehensive measurement data on weld seams. Such data form the basis for the employment of Deep Learning approaches to interpret and evaluate measurement signals from inline process monitoring systems in laser material processing. The procedure and the potentials are illustrated in Figure 10 using the example of the holistic utilization of OCT keyhole depth measurements. With the knowledge about the weld properties at each point of a measurement signal, the signals of the sensor can be characterized precisely and used for the further development of welding process models [26]. In addition, ray-tracing approaches that correlate the keyhole geometry with measurement signals can be validated [27]. The results thus indicate excellent potential for the prediction of weld seam features starting from inline measurements of the keyhole depth.

## Figures and Tables

**Table 1:** Materials investigated based on µCT within the preliminary investigations.

Alloy	Heat Treatment	Alloying Elements (Percent by Weight)
AA 2219	T87	Cu 6.3; Mn 0.3; Ti 0.06; V 0.1; Zr 0.18
EN AW-5083	—	Mg 4.4; Mn 0.7; Cr 0.15
EN AW-5083	artificially aged ^1^	Mg 4.4; Mn 0.7; Cr 0.15
EN AW-6060	—	Si 0.4; Mg 0.5; Fe 0.2
EN AW-6082	—	Si 1.0; Mg 0.85; Mn 0.65

^1^ The parameters of the artificial ageing process were not available from the manufacturer; the material EN AW-5083 was not further considered after the preliminary tests, however.

**Table 2:** Measurement parameters of the µCT evaluation and resulting measuring dimensions.

Resolution	Voxels	Dimensions
7.7 µm	700 × 700 × 1600	5.39 × 5.39 × 12.32 mm³
10.0 µm	600 × 600 × 1600	6.00 × 6.00 × 16.00 mm³
15.0 µm	400 × 400 × 1700	6.00 × 6.00 × 25.50 mm³
17.0 µm	320 × 320 × 800	5.44 × 5.44 × 13.60 mm³
